# An Empirical Study on the Promotion of Students’ Physiological and Psychological Recovery in Green Space on Campuses in the Post-Epidemic Era

**DOI:** 10.3390/ijerph20010151

**Published:** 2022-12-22

**Authors:** Ping Zhang, Qianyi He, Zexuan Chen, Xi Li, Jun Ma

**Affiliations:** College of Landscape Architecture, Sichuan Agricultural University, Chengdu 611130, China

**Keywords:** visual perception, restorative effects of campus landscape environment, landscape types, landscape elements

## Abstract

Human health is closely related to the environment; a relaxing and pleasant landscape environment can make people feel less stressed and more energetic. To investigate the restorative potential of landscape types and landscape elements in the post-epidemic era from the perspective of visual perception, this study selected Sichuan Agricultural University’s Laoban hill, Jiuqu bridge, and the ginkgo garden to carry out physiological and psychological measurement experiments with college students. Research results on the psycho-biological and perceptual recovery vary with the types of landscape spaces. The results of the physiological data showed that all three space types had no significant effect on the recovery of blood pressure and heart rate; Laoban hill and Jiuqu bridge had some impact on concentration, while the ginkgo garden had no significant effect; and all three space types had some effect on the relaxation of the subjects’ mental state. The results of the psychological data showed that the subjects’ emotions were significantly improved in the three different landscape space types. The space with the strongest restorative effect on negative emotions was the ginkgo garden, followed by Jiuqu bridge and Laoban hill. The spaces with the strongest restorative potential for positive emotions were Jiuqu bridge and the ginkgo garden, followed by Laoban hill. The results of the perceptual restoration data showed that the Laoban hill space had the most effective restorative potential on the human body, followed by Jiuqu bridge, with the ginkgo garden having the least effective restorative potential. The results of the study on the difference between the aesthetic preference of different landscape elements and the perception restorative effect of a space showed that “the harmony between artificial structures such as garden pieces and the environment”, “plant species”, “waterscape state”, and “boundary clarity” were identified as significant landscape elements with perception-restorative effects. These findings summarize campus landscape types and elements with optimal restorative potential. In the future, in campus landscape design—an active approach with a scientific combination and configuration of campus landscape types and elements—can provide a feasible solution to enhance the potential of campus landscape restorative effects.

## 1. Introduction

Rapid urbanization causes a series of health problems when creating enormous economic value. In its latest State of the World’s Cities Report 2022, UN-Habitat states that more than 56% of the population in the world currently lives in urban areas, and that this proportion will rise to 68% by 2050, leading to a range of socio–environmental issues as the urban population proliferates (World Cities Report 2022) [1]. Fast-paced, high-stress, and crowded urban living spaces far from the natural environment are key factors in causing physiological diseases, such as obesity and cardiovascular disease, and mental diseases, such as anxiety and depression [2]. The acceleration of the social rhythm, the fierce competition in academia and job searching, and the impetuousness of the information explosion have dramatically increased the pressure on college students. The incidence of morbidity of mental illness is also constantly rising. Stress has become a major factor affecting the physical and mental health and well-being of college students [3]. Since the outbreak of COVID-19 at the beginning of 2020, dramatic changes have been observed in people’s lifestyles. University campuses feature a high-density population, which requires strict control of campus access to reduce the infection risk of college students exposed to public spaces. According to previous literature, interview results show that long-term school-closure management has led to a sharp increase in the mental stress of college students. College students need a close-to-nature campus space to relieve their mental stress. In the epidemic area, the psychological survey data on college students show that the anxiety and depression incidence rates in college students is higher than that of the general population [4]. During the epidemic lockdown, campus provided a limited green space for college students. A campus is a public environment where students can experience the highest degree of exposure to a green space during the epidemic. A healthy campus landscape space can effectively relieve students’ psychological stress. However, at present, the land in main urban areas tends to be constricted. Additionally, in order to maximize the teaching function of a campus, the construction of university campus landscape space has many defects in its style and spatial simplicity and lacks the sufficient beauty and naturalness which would relieve negative emotions such as tension and anxiety among college students.

Humans who get close to nature receive physical and mental restorative effects from nature. The natural environment is considered an important factor for physical and mental restoration [5]. People are more liable to think positively through self-regulation and self-reflection to achieve mental recovery in an environment which they like and feel safe and comfortable in. [6,7]. Studies have shown that green environments have a higher restorative effect than built environments. Exposure to green environments is associated with a greater positive mood, cognitive performance, and increased physical health, compared to urban or built environments. It has also been confirmed that natural spaces have more significant restorative properties than urban green spaces with a significant proportion of artificial architecture [8,9,10]. Recent studies have shown that differences in natural landscape types have significantly different impacts on people’s aesthetic preferences and their perceptions of its restorative effects [11,12,13,14,15,16]. The best restorative spaces are natural spaces, followed by waterscape spaces, with spaces for sports and activity being the least restorative spaces. This proves that natural spaces have a more significant restorative effect than urban green spaces that are obviously artificial. For tired college students, being healed by nature is the main behavioral motivation for visiting green spaces. Creating campus green landscape spaces can increase students’ opportunities intentionally, accidentally, or indirectly to be exposed to nature and social activities, which have remarkable up-regulated effects on college students’ psychological restoration. Some experts have delved into the role of specific landscape elements in restoring and promoting mental health [17,18,19]. The composition of elements related to nature (bamboo forests, grasslands, shrubs, lakes, and flowers) positively correlates with restorativeness to a significant degree. Among campus landscape spaces, spaces with high vegetation coverage and hydrophilicity are more conducive to the spiritual recovery of highly stressed individuals. Most of the artificial elements (e.g., planting beds, soil retaining, and landscape lights) are not restorative, and the hard paving of roads and plazas can even have a negative impact [20,21].

Based on the above findings, this study intends to further explore the differences in physical and psychological recovery and the perception-restoration of college students exposed to different types of campus green spaces, as well as the interaction between aesthetic preferences for campus landscape elements and the perception spatial recovery under different types (the same landscape elements may provide different experiences to subjects in different types of spaces [19]). Our group will identify the types of spaces and corresponding landscape elements that specifically affect the health and restoration of the population. This study intends to address the following questions:

(1) How do different types of green spaces on campus differ in terms of their physical and psychological restoration for college students?
(2) What are the differences in the perception-restorative effects for college students of different types of green spaces on campus?
(3) What is the relationship between the aesthetic preference of landscape elements and the perception-recovery of space under different kinds of landscape on the campus?

## 2. Materials and Methods

### 2.1. Participants

Thirty native Chinese student volunteers (fourteen males and sixteen females, aged 19–20) were recruited from the Chengdu campus of Sichuan Agricultural University in three majors: landscape architecture, gardening, and environmental design. Volunteers were instructed to avoid smoking, consuming alcohol, and engaging in vigorous physical activity throughout the study. According to information from pre-experimental interviews, volunteers generally chose green spaces for relaxation. The choice of campus green-space environment can be influenced by factors such as spatial scale, privacy, degree of greenery, accessibility, and leisure time. In order to ensure the accuracy of the experiment, the experimental sites were located on two other campuses (Ya’an Campus and Dujiangyan Campus) where the volunteers do not live or study for a long time. These locations had almost the same climatic conditions. The volunteers had the same degree of unfamiliarity with these two campuses. 

### 2.2. Study Sites

Based on the research data from the environment, combined with the eight perceptual dimensions that can be used to describe the characteristics of different landscape environments proposed by Grahn and Stigsdotter, the three locations of this experiment were selected; namely: Laoban hill, Jiuqu bridge, and the the ginkgo garden. These locations represented three typical features of campus landscape spaces in the urban natural environment [22].

The landscape space type of Laoban hill is a natural mountain forest (low hill). Its main landscape elements included “autochthonous and ornamental forests (*Cupressus funebris* Endl., *Ginkgo biloba* L., Bambusoideae, *Rhapis excelsa (Thunb.) Henry ex Rehd.*, *Broussonetia papyrifera* (Linnaeus) L’Heritier ex Ventenat, *Alangium chinense* (Lour.) Harms, *Nandina domestica* Thunb., etc.)”, “flowers”, “stones”, “streams”, “viewing platforms”, “roads”, “trails”, “pavilions”, “steps”, “wooden seats”, etc. The elevation of this area is about 600–750 m, with more than 90% green coverage. It has a high environmental depression and undulating terrain. Laoban hill is far away from the daily study life of university students and can provide a quiet, natural environment, which is defined as a natural mountain space with natural and tranquil characteristics. The landscape space type of Jiuqu bridge is a Chinese classical garden. Its main landscape elements are “bridge”, “lake”, “pavilion”, “corridor”, “plants (Salix babylonica, Bambusoideae, *Nelumbo nucifera* Gaertn., etc.)”, “paths along the lake”, “lamp posts”, etc. The semi-open characteristics of Jiuqu bridge, with a flat terrain and water scenery, mainly meet the needs of college students for privacy and security. It defined as a cultural space with the function of a shelter. The ginkgo garden features foreground social space. Its main landscape elements are: “lawn”, “sculpture”, “scenic wall”, “stone”, “flower bed”, “pavement”, etc. The lawn area in the middle is enclosed by regular and uniform plants (such as *Ginkgo biloba* L., *Metasequoia glyptostroboides* Hu & W. C. Cheng, *Phoebe zhennan* S. Lee et F. N. Wei, etc.) and seats under the trees, while the periphery is a road. As it is the main circulation space of the campus, the topography is very flat, and the passage paths are symmetrical and clear with high elasticity. The layout of trees on the boundary of the site is regular and uniform. The ginkgo garden is the main passage space of the campus, with an open lawn where students can observe the surrounding environment and facilitate social activities. It was defined as a prospect and social space.

In addition, the experimental plots have a low external impact on the experiment (no bikes or cars in visible sight). As a natural mountain, the area of Laoban hill is much larger than that of the other two sites, but the space used in the test was not significantly different from the other two spaces. Detailed information about the three study sites is shown in Table 1.

### 2.3. Experimental Design

The experiment mainly explored the differences in the recovery effects of different landscape types and landscape elements on college students from the data concerning physiology, emotion, perception, and preference. The three experimental plots were situated at two campuses (Ya’an Campus and Dujiangyan Campus; both are not the campuses where the volunteers study and live for a long time). Volunteers were first sent to the Ya’an campus to participate in the experiment. They were subsequently sent to the two other two experimental sites at the Dujiangyan campus. The interval between the three spatial experiments was more than two hours, which can eliminate legacy effects. Participants were randomly divided into two batches for the experiment, with an interval of one week. The experimental temperature (16 °C ± 1 °C and 15 °C ± 1 °C) and air humidity (96%, 94%; 99%, and 95%) were similar, eliminating the influence of environmental factors on the experiment. The two batches were kept at the same experimental time (9:30–11:30 am and 1:30–6:10 pm) to eliminate the influence of diurnal variation in physiological rhythms. 

**Figure 1:**
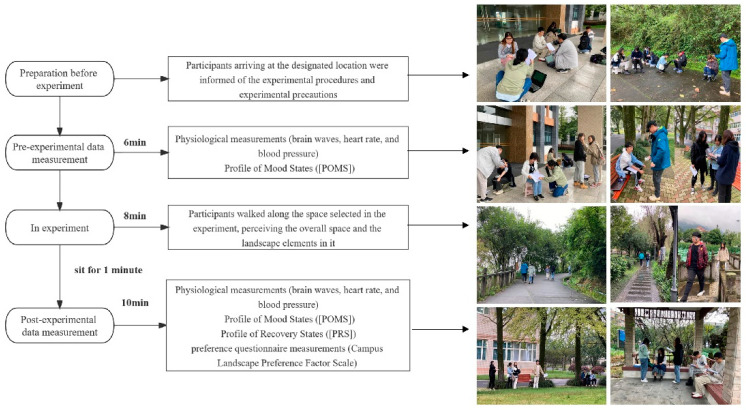
The Experimental Produce.

The point of departure of all the participants was appointed to the same site, and the destination was Laoban hill. After arriving at Laoban hill, participants were informed about the experimental process and experimental precautions. They were then divided into four groups to enter the experiment in turn. The first step was a pre-experimental measurement: participants rested in a seated position for 6 min (it has been shown that a 5-min experience is long enough to produce a complete recovery effect [23]) in a designated area while taking both physiological measurements (brain waves, blood pressure, and heart rate) and emotional state measurements (Profile of Mood States, POMS). Next, the participants walked along the selected space for eight minutes under the guidance of the assistants, while the other participants waited in turn according to their group. During the walking process, the participants needed to perceive the overall space and the landscape elements. In the third step, the participants were brought to the designated resting place to sit for 1 min to ensure that the experimental participants had a similar physical-fatigue degree to what they demosntrated before taking the measurements. Physiological measurements (brain waves, heart rate, and blood pressure), Profile of Mood States (POMS), Profile of Recovery States (PRS), and preference questionnaire measurements (Campus Landscape Preference Factor Scale) were then completed under the guidance of an experimental assistant. The experimental procedure remained the same throughout the whole process. Figure 1 shows the experimental procedure.

### 2.4. Measurements

During the experiment, a sphygmomanometer and a portable electroencephalograph were used for physiological measurements, a POMS scale was used for emotional state measurements, a PRS scale was used for perception-recovery measurement, and a scale of campus environment perception preference factors was used for the preference questionnaire measurement. This study explored differences in physiological indicators, emotional states, perception recovery, and preferences among different types of landscape spaces and landscape elements. A total of thirty valid experimental data samples were collected in the experiment.

#### 2.4.1. Physiological Measurements

Before and after the whole experiment, blood pressure (systolic, mmHg; diastolic, mmHg; and pulse rate, bpm) was measured in the left arm using a sphygmomanometer (OMRON, HEM-8102A, Dalian, Liaoning, China), which was regarded as an indicator of the body’s state of arousal or relaxation. The brain waves were recorded using a portable EEG device (Sichiray, 03, Wuxi, Jiangsu, China) within 120 s of the interval values in the attention level and relaxation level of the participant’s brain, reflecting the influence of the environment on the participant’s brain relaxation and attention. The parameters of attention and relaxation were both indicated by specific values between 1 and 100 at intervals of 20. The parameter of attention indicates the brain concentration level of the participants, while the parameter of relaxation indicates the participants’ current mental states. 

#### 2.4.2. Emotional State Measurement

This study used the Profile of Mood States [POMS] and the Perceived Restorativeness Scale [PRS] to evaluate participants’ psychological measurements. The POMS questionnaire is shown in Appendix A Table A1. It consists of forty questions to assess the emotional fluctuations of individuals, which are divided into five negative emotional sub-scales including “tension–anxiety (T–A)” “depression–discouragement (D)”, “anger–hostility (A–H)”, “fatigue–sluggishness (F–S)”, and “flustered–confuse (F–C)”; and two positive emotional sub-scales including “energy–vitality (E–V)” and “self–emotion (S) [24].” The answers to each question were scored from 0 to 5. The higher the score, the higher the emotional level. The overall mood disturbance degree (TMD) was obtained by adding the negative mood sub-scales, subtracting the positive mood sub-scales, and adding 100 points.

#### 2.4.3. Perceptual Recovery Measurements

The PRS questionnaire is shown in Appendix A Table A2. According to the experimental requirements of this research, the PRS questionnaire was composed of nine items, including four dimensions: Being Away, Extent, Fascination, and Compatibility [25,26,27]. Participants were able to score on a five-point scale ranging from 1 (totally not strong) to 5 (very strong).

#### 2.4.4. Preference Questionnaire Measurements

The questionnaire adopted the self-designed Campus Environment Perception Preference Factor questionnaire, shown in Appendix A Table A3. The aesthetic preferences of college students on different landscape elements were discussed through the preference selection of landscape elements of different landscape types. The parameters of the questionnaire were mainly composed of the following elements: natural restorative environment elements, artificial restorative elements, and psychological perception restorative elements [28]. The elements of a natural restorative environment include plants, water, and terrain; the elements of artificial restoration are composed of recreational facilities and landscape architectures; and the elements of psychological perception and restoration are social and spatial psychological perception.

### 2.5. Statistical Analyses

To determine the differences between physiological and psychological restorativeness in the three landscape types, paired *t*-tests were employed to analyze the POMS score and the mean values of the physiological data. A one-way ANOVA was used to analyze the differences between the scores of PRS and dimension. The relationship between the restorative perception of three landscape types and the aesthetic preference of landscape elements was explored using Pearson correlation analysis by sorting out the landscape elements significantly correlated with the restorative perception and then using the stepwise multiple linear regression analysis methods to identify the critical landscape elements that affected the restorative perception of the campus. The statistical analysis was performed with SPSS 26.0 (SPSS Inc., Chicago, IL, USA), and a *p*-value < 0.05 was considered to indicate statistical significance. The results of the resulting data are presented via GraphadPrim’s 7.0 plots.

Considering the multi-collinearity among landscape elements in each space, we used a stepwise multiple linear regression analysis to establish a quantitative relationship between all significant perceptual restoration factors and landscape element preferences. When the value of tolerance <0.2 or VIF > 10 in the stepwise multiple linear regression analysis indicated the presence of covariance among the independent variables, the generated equation model was unreliable. The models in this study are well accepted and have no problems with multicollinearity (minimum tolerance value = 0.789 and maximum VIF = 1.267 in Table 2; minimum tolerance value = 0.753 and maximum VIF = 1.327 in Table 3; minimum tolerance value = 0.962 and maximum VIF = 1.040 in Table 4).

## 3. Results

### 3.1. Restorative Differences in Different Landscape Types in Campus Environments

#### 3.1.1. Physiological Results

**Figure 2:**
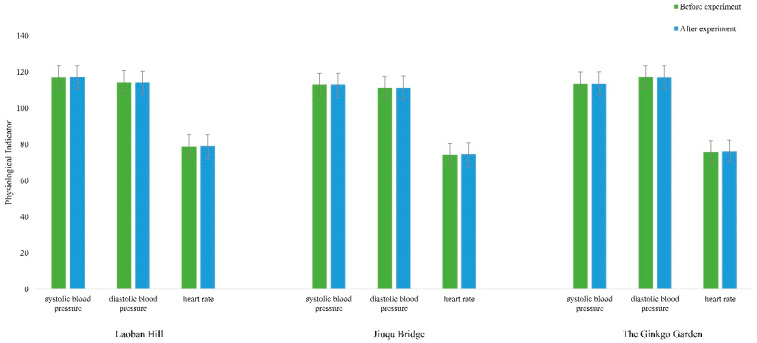
Blood Pressure and Heart Rate.

In the experiment on Laoban hill, Jiuqu bridge, and the ginkgo garden, no significant changes were observed in the participants’ diastolic blood pressure, systolic blood pressure, or heart rate (Figure 2). 

**Figure 3:**
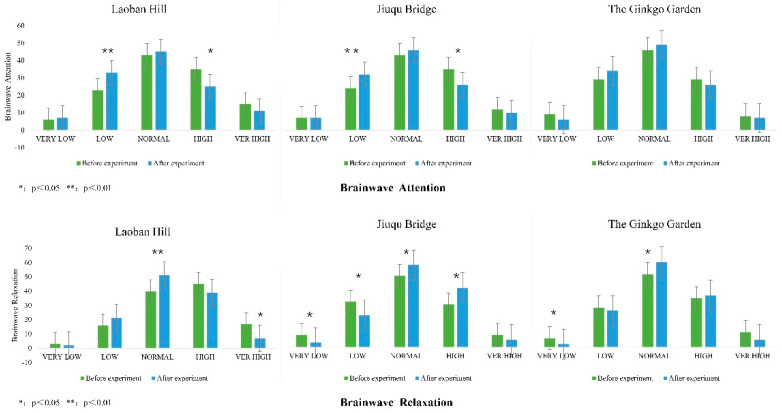
Brainwave Attention and Relaxation.

In the brainwave concentration data (Figure 3), before and after the experiment, the value of Laoban hill was significantly different between 21–40 (lower) (*p* < 0.01) and 61–80 (higher) (*p* < 0.05). Jiuqu bridge showed significant differences; 21–40 (lower) (*p* < 0.05) and 61–80 (higher) (*p* < 0.05), and the ginkgo garden was not significant. Before and after the Laoban hill and Jiuqu bridge experiments, the lower level of concentration times increased, and the higher level of concentration times decreased. There was no change in the data before and after the ginkgo garden experiment. The three spaces did not have a better recovery effect on concentration.

In the brainwave relaxation data (Figure 3), before and after the experiment, the value of Laoban hill had a significant change in the range of 41–60 (general) (*p* < 0.01) and 81–100 (higher) (*p* < 0.05). Jiuqu bridge demonstrated significant changes in the range of 0–20 (very low) (*p* < 0.05), 21–40 (lower) (*p* < 0.05), 41–60 (general) (*p* < 0.05), and 61–80 (high) (*p* < 0.05). The ginkgo garden also showed significant changes in the range of 0–20 (very low) (*p* < 0.05) and 41–60 (general) (*p* < 0.05). Before and after experiments, the lower level of relaxation time decreased, the general level of relaxation time decreased, and the higher level of relaxation time decreased. There was no change in the data before and after the ginkgo garden experiment. The three spaces did not have a better recovery effect on relaxation.

#### 3.1.2. Psychological Results

**Figure 4:**
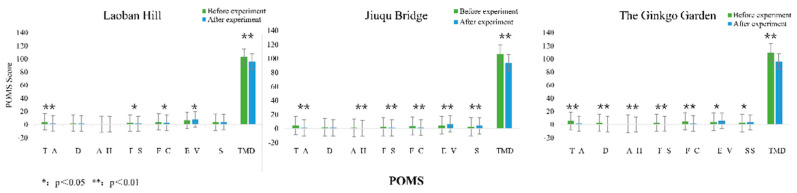
POMS Scale.

The results of the POMS scale are shown in Figure 4. At Laoban hill, participants’ scores on the three negative emotion components, “tension–anxiety (T–A) (*p* < 0.01)”, “fatigue–sluggishness (F–S) (*p* < 0.05)”, and “flustered–confused” (F–C) (*p* < 0.05)” showed a significant decreasing trend. The positive emotion component, “energy–vitality (E–V) (*p* < 0.05)”, showed a significant increasing trend, and the overall mood-disturbance degree (TMD) (*p* < 0.01) also showed significant differences before and after the test.

At Jiuqu bridge, participants’ scores on the four negative emotion components, “tension–anxiety (T–A) (*p* < 0.01)”, “anger–hostility (A–H) (*p* < 0.01)”, “fatigue–sluggishness (F–S) (*p* < 0.01)” and “flustered–confused (F–C) (*p* < 0.01)”, showed a significant decreasing trend. The positive emotion components “energy–vitality (E-V) (*p* < 0.05)” and “self-emotion (S) (*p* < 0.01)” showed a significant increasing trend, and the overall mood-disturbance degree (TMD) (*p* < 0.01) also showed significant differences before and after the test.

In the ginkgo garden, participants’ scores on all negative emotion components, “tension–anxiety (T–A) (*p* < 0.01)”, “depression–discouragement (D) (*p* < 0.01)”, “anger–hostility (A–H) (*p* < 0.01)”, “fatigue–sluggishness (F–S) (*p* < 0.01)”, and “flustered–confused” (F–C) (*p* < 0.01)” showed a significant decreasing trend. The positive emotion components “energy–vitality (E–V) (*p* < 0.05)” and “self-emotion (S) (*p* < 0.05)” showed a significant increasing trend, and the overall mood-disturbance degree (TMD) (*p* < 0.01) total scores also showed significant differences before and after the test.

From the above results, it can be found that the space with the strongest restorative effect on negative emotions was the ginkgo garden, followed by Jiuqu bridge and Laoban hill. The spaces with the strongest restorative effect on positive emotions were Jiuqu bridge and the ginkgo garden, followed by Laoban hill. Participants’ emotions were significantly improved in different types of landscape spaces.

**Figure 5:**
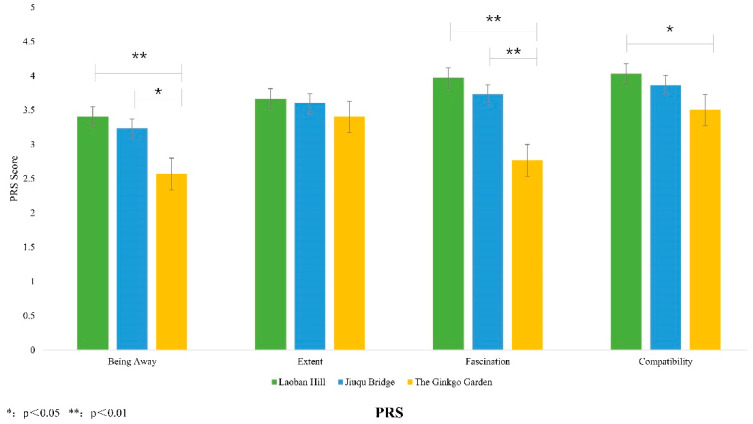
PRS Scale.

The PRS results are shown in Figure 5. The Being Away score of Laoban hill (3.40 ± 1.003) was significantly different from the ginkgo garden (2.57 ± 0.774) (*p* < 0.01), and Jiuqu bridge (3.23 ± 1.194) was significantly different from the ginkgo garden (2.57 ± 0.774) (*p* < 0.05). There was no significant difference between Laoban hill and Jiuqu bridge. Regarding the Extent score, none of the three spaces were significantly different from each other. The Fascination score indicated that Laoban hill (3.97 ± 0.89) and the ginkgo garden (2.77 ± 1.04) had significant differences (*p* < 0.01), and Jiuqu bridge (3.73 ± 0.944) and the ginkgo garden (2.77 ± 1.04) also had significant differences (*p* < 0.01); there was no significant difference between Laoban hill and Jiuqu bridge. The Compatibility score indicated that Laoban hill (4.03 ± 0.809) and the ginkgo garden (3.50 ± 0.938) had significant differences (*p* < 0.05), and there was no significant difference between Jiuqu bridge and the ginkgo garden and no significant difference between Laoban hill and Jiuqu bridge.

### 3.2. Differences in Aesthetic Preference and Restorative Perception of Landscape Elements in Campus Environments

By filling in The Campus Landscape Preference Factor Scale, which explored students’ aesthetic preferences for different landscape elements, correlation analysis was carried out with the perception restoration of different landscape types to obtain the most restorative landscape elements in different landscape types.

The results are shown in Table 2, Table 3 and Table 4. “The view probability of the lounge seats”, “stepped shade coverage”, “cultural character of the landscape wall”, and the “harmony of garden sculpture with the environment” had a positive impact on the restorative perception of Laoban hill. The “gentle slope of the trail”, “arbor species”, and “flowering season” had negative effects on the restorative perception of Laoban hill. “The state of the water”, “landscape lamp post height”, and the “harmony of the garden pavilion and environment” had a positive impact on the restorative perception of Jiuqu bridge. The “proximity between garden bridge and plants”, “the proximity between the garden bridge and water”, the “climbing plant species”, “the layer of aquatic plants”, and the “landscape lamp post light color” had a negative impact on the perception of restoration of the space of Jiuqu bridge. “The clarity of boundary”, “harmony of landscape stone with the environment”, and “the probability of staying in the site” had a positive impact on the restorative perception of the ginkgo garden. The “arbor planting density “and “distribution of rest seats” had negative effects on the restorative perception of the ginkgo garden.

On Laoban hill, the landscape element that had the greatest impact on the perception of restorativeness was the harmony between sculpture and environment, followed by the species of trees; at the Jiuqu bridge, the landscape element that had the greatest impact on the perception of restorativeness was the species of climbing plants, followed by the state of waterscape; the landscape element that had the greatest impact on restorative perception in the ginkgo garden was the boundary clarity, followed by the landscape stone placement and environmental harmony. These landscape elements were shown to be significantly associated with restorativeness.

## 4. Discussion

### 4.1. Different Effects of Landscape Types in the College Students’ Psychological Relaxation and Perception Recovery

This study discusses the differences in restorativeness of three different landscape types by comparing the data of physiological indicators, emotional states, and the perception recovery of participants’ exposure to the three different landscape types. There is no significant change in the three types of space for blood pressure as well as heart rate data before and after the experiment. Additionally, there is also no significant recovery effect on the concentration and relaxation of brainwaves. This may be because individuals can become physiologically restored rapidly. This can start to affect physiological responses in less than four minutes, reaching physiological recovery in a relatively short period of time, and this effect can last for a longer period [29,30]. All three natural environmental spaces contribute to removing negative emotions and promoting positive emotions. The space with the strongest restorative effect on negative emotions is the ginkgo garden, and Jiuqu bridge and the ginkgo garden share the strongest auxo-action on negative emotions. The three types of landscape spaces have significant differences in perception recovery for college students.

The results indicate that Laoban hill, as a space of nature and serenity, has the best perception-recovery effect, but a weaker effect on mental recovery. According to the eight perceptual dimensions proposed by Grahn and Stigsdotter, the most favorite space of the population is a space of nature and serenity [22]. This kind of space is far away from the dense building clusters and communities where one could experience the charm of nature to obtain peacefulness and tranquility. Numerous studies have proven that the natural environment has better restorative effect than the artificial natural environment, and that the woodland landscape has the best relaxation effect [25,31]. As important landscape elements in the restorative landscape, dense flora in woodlands can provide comfortable sensory stimulation to the human body and improve the relaxation of human body in the natural environment, as well as play a more positive role in promoting health [32]. At the same time, the rich topographic changes in the woodland landscape provide the environment with a space of privacy and security, causing people to enjoy tranquility and solitude, achieve psychological relaxation, and enhance happiness [22,33]. 

Laoban hill, a green space away from daily life, is not as accessible as the other two spaces. This makes it a space that stressful college students would choose to visit when they have more leisure time. Although Laoban hill itself has a higher quality of green environment than the other spaces, it is not the best choice for college students who need to reduce stress due to its relatively poor accessibility, thus weakening the emotional recovery for the crowd [34]. Tina Gerstenberg and Mathias Hofmann proposed that a space with dense trees may come at the price of decreasing accessibility and visual penetration through a stand, which is negatively associated with perception safety [35]. Additionally, dense forest spaces do not always provide a high-quality experience; mosquitoes or other microorganisms in the forest may have other effects on the population, thus reducing their emotional restoration for college students [36].

Jiuqu bridge has a stronger effect on positive emotional recovery than Laoban hill and a weaker effect on negative emotional recovery than the ginkgo garden. It ranks in the middle for its effect on perception recovery. Jiuqu bridge is a cultural space with the function of a refuge and a humanistic space that can characterize Chinese classical gardens. According to the research results of Grahn and Stigsdotter, culture space, as the essence of human civilization in space, contains the cultural spirit of Chinese classical gardens [22]. By artificially creating a space that resembles a natural environment, we can create an artistic conception with special cultural connotations that can trigger a spiritual experience beneficial to physical and mental health [37]. As a waterscape space, Jiuqu bridge has an obvious relaxing effect on the human body [11]. As a semi-open waterscape space with the function of a refuge, the space provides people with a sense of sight-encirclement and meets people’s needs for privacy and security [22,38]. Additionally, the bridge satisfies the theory of “prospect and refuge” [39]. The space reflects the dialectic philosophy of the “unity of man with nature” in Chinese classical gardens. It can significantly promote people’s physical and mental health by highlighting the harmony and balance between natural landscapes such as waterscapes and plants and human landscapes such as pavilions [19]. 

Between the three spaces, the restoration effects of the ginkgo garden are inferior to those of Laoban hill and Jiuqu bridge, but its effect on relieving the negative emotions and enhancing the positive ones is superior to the other two places. The ginkgo garden is a prospect space for social interaction. It is an open-plan area with a well-kept lawn and a clear view. Such spaces with foreground and social characteristics are usually used for activities or entertainment with the worst restorative effect [11]. This landscape type can meet people’s needs for “prospect” in the environment, but not for “refuge.” This is due to the excessive opening of the space, which undoubtedly increases the mental pressure of the participants. A person affected by stress instinctively will seek out places to hide and finds it increasingly difficult to socialize with others, but such spaces do not satisfy such requirements [40,41]. High-stress people, such as college students, are more likely to have positive emotions when staying alone in a relatively hidden space [33]. As an open, pedestrian-oriented circulation space, the ginkgo garden lacks a sense of privacy and solitude. Compared to spaces composed of natural vegetation and bodies of water, the ginkgo garden has an overly high density of pedestrian traffic, which also affects the restorative effect of the environment for people [29].

However, as a space that exists for social activities, the green space improves mood by providing activities that promote social contact and interaction, thereby strengthening the sense of community belonging as well as increasing community harmony and cohesion [42]. The high accessibility of the ginkgo garden space indicates its proximity to daily life, making it the type of space that college students can come into frequent contact with. It has become green space where students choose to stop, experience nature, and obtain a short break even under pressure of daily work. Improving the accessibility and reachability of green space is beneficial to emotional recovery to a certain extent [34]. For people with short rest and relaxation periods, such a space can provide free mindfulness training and effectively promote mental restoration.

As was indicated by the restoration differences of three typical landscape types different landscapes are associated with different levels of restorativeness on college students’ mental health and perception stress. This further confirms that space plays an important role in helping people become perception-restored as well as relieving their fatigue. 

### 4.2. Aesthetic Preference and Environmental Restoration Potential of Different Landscape Elements

In this study, we measure and evaluate participants’ aesthetic preferences for landscape elements in three types of landscape spaces and conduct an analysis of the correlation between the aesthetic preferences of landscape elements and the restorative perception of landscape types.

Restorative features of environmental perception are positively correlated with aesthetic preferences and positive emotions [43]. A person’s aesthetic preferences may play a crucial role in the experience of relaxation and stress relief [44], and there is a positive relationship between the preference for a particular environment and the environment’s restoration potential for stress or mental fatigue [45]. Restorative experiences occur when people are in their preferred environment, which contributes to emotional development [6]. The more preferred the environment is, the more relaxed and happy people can become from the environment, releasing pressure, which can improve their mental health [46]. The research results show that the landscape elements significantly related to restoration are “the harmony between artificial structures such as garden pieces and the environment”, “plant species”, “waterscape state”, and the “boundary clarity.”

In previous studies, artificial elements have referred to a negative effect on aesthetic preferences and perception recovery [47]. However, this study finds that the more harmonic relation the artificial elements have to the environment, the stronger the restorative effect the environment will be. As a part related to culture and art, garden sketches (e.g., landscape sculpture or landscape setting stone) are an important source of discovering new things and exercising imagination. They can be used as a means of stimulating thinking and have an obvious positive effect on mental recovery [19]. This supports the findings that cultural heritage and art sites are frequently perceived to have high levels of restorative properties [48]. In the Gestalt Theory, “the law of the relationship between the figure and the ground”, “it is believed that the key element expressed in the environment is the ‘figure’, and the background that acts as a foil around the figure is the ‘ground.’” The greater the difference between “figure” and “ground” is, the easier it is to perceive [49]. With the natural green forest as the background of Laoban hill, the “figure” of the artificial element forms a strong contrast with the “ground” of the green natural forest map, which may unconsciously attract people. While retaining the relationship between artificial elements and the natural environment, enhancing the harmony between the two can enhance the attraction and charm of the space. Additionally, it can also promote the attention and attraction of participants. This is also in line with the views of previous scholars that landscape elements in different landscape-type environments can bring different feelings to the experiencer. In this way, landscape elements in the environment cannot be considered absolutely or in isolation.

This study proves that spaces with limited plant species arranged in a harmonic way are positive restorative landscape elements. This kind of space features good ductility, where the plantscape can be formed naturally and people can feel less stressed in such a harmonic environment. Relatively neat pattern of plants can offer people a wider view than the chaotic and rough spatial patterns, and people are more likely to feel relaxed and pleased [50]. Plant species have been shown to affect environmental restorative effects by affecting plant appearance and color [51,52]. Green plants can make people have a relaxed mood and show more positive emotions. Natural green spaces positively impact people’s psychological recovery [30]. In the selection and make-up of trees, the identified perceptual tree parameters enable the selection of different tree species which look similar, resulting in the creation of a coherent overall picture with a simultaneous increase of species diversity in urban areas, which increases the resilience of tree populations to pests and diseases [35]. 

The space mainly formed by the waterscape can be used as a healing place to endow college students with positive mental health and has a positive impact on the restorative effect of the environment. Static water features have a vast and calm water surface that can mirror the sky and surrounding environment, making it easier for people to fall into meditation and contemplation to achieve the effect of psychological restoration [27,53]. At the same time, the static waterscape can provide people with a quiet space so that college students are more willing to do what they like in this environment to satisfy their individual needs. A waterscape combined with plants creates a micro-climate environment, which can improve the comfort of the site and form a space with more attractive features. This type of space is very attractive to college students who need to release their mental pressure. They can explore and experience the landscape without spending too much energy and gain a more relaxing and calming experience [54,55].

In addition to the objective characteristics of landscape elements, the spatial characteristics of subjective perception also have an impact on recovery. The clearer the spatial boundaries are, the easier it is to have a positive restoration effect on people’s biological psychology. Greenfield environments do not always result in a high-quality perceptual restorative experience. It seems that spaces with dense plants but without clear boundaries may become a hiding place for potential attackers. People cannot observe the overall space because of plant-bound vision, which enhances people’s sense of fear and crisis, consumes more direct attention, and is not conducive to human recovery for health [35,56]. People are more inclined to stay in places with clear boundaries where they can have a wide view to observe abundant spatial contents, satisfying people’s need for a sense of security in the space [57,58,59]. The safety need is the most basic necessity of human survival and behavior [60]. Only if college students obtain the necessary sense of security is there a chance for them to become relaxed to some degree.

## 5. Conclusions

Historically, urban public health safety has always been an important part of urban planning. Thanks to the development of modern medicine and the improvement of living standards and the environment, better conditions have been provided to reduce the incidence of some infectious diseases [61]. However, with the development of urbanization, people are gradually moving away from the natural environment, which increases the incidence of mental diseases such as depression. People are in urgent need of more access to nature and more green spaces for restoration. Green spaces on college campuses play an important role in the health recovery of college students.

From the perspective of college students’ visual perception, this study explores the different effects between the different landscape types on campus and the different landscape elements on college students’ health and perception restoration. It was demonstrated that different landscape types and landscape elements have different restorative effects. Laoban hill, as a space of nature and serenity, may provide the best restorative experience; Jiuqu bridge is a cultural space that also serves as a refuge; and the ginkgo garden may provide the poorest restoration as a space for prospective social interaction. “The harmony between artificial structures such as garden pieces and the environment”, “plant species”, “waterscape state”, and “boundary clarity” are important restorative attributes that can enhance the restorative environmental benefits and effectively promote college students’ physical and mental health. Based on the conclusion of this study, the construction of the university campus landscape should focus on alleviating mental fatigue and stress and promote relaxation among students. Campus green spaces and reasonably configured spatial features and landscape elements are fully used to build a good campus environment.

This study has certain limitations. It explores the restorative differences of the environment from the perspective of visual perception. In future research, more perceptual factors can be added to study the restorative differences, such as the restorative differences of different types of perceptual factors and combinations of different types of perceptual factors. In the measurement of the restorative environment in colleges and universities, based on the need to control the external objective environmental variables and the restriction of population mobility caused by the impact of the epidemic, the sample size of this experiment was only selected as a target group with the most typical high-stress college students, and the sample size is relatively limited. However, there are postgraduates, doctoral students, and faculty members among high-stress groups on university campuses. It is important to determine whether people of different ages and educational levels have a different impact on the results of the study, and whether there would be different students’ psychological relaxation results and aesthetic preferences. Therefore, the types and number of participants need to be enriched in future studies.

## Figures and Tables

**Table 1:** Descriptions of Three Study Sites.

Site	Laoban Hill	Jiuqu Bridge	The Ginkgo Garden
Mapping	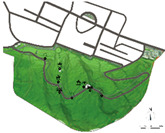	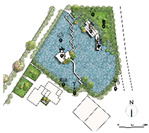	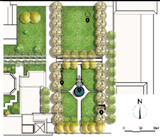
Site Area	S = 158,250 m^2^	S = 5676 m^2^	S = 4746 m^2^
Images	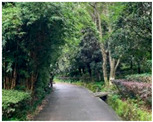	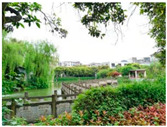	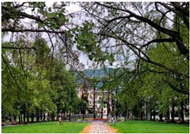
	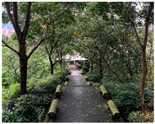	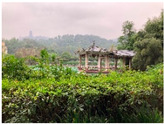	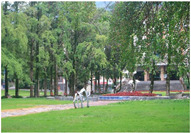
	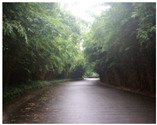	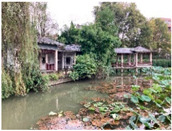	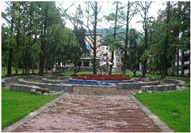
	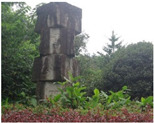	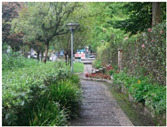	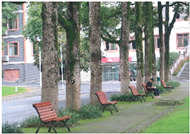
Spatial Types	A natural and serene space.	A cultural refuge space.	A foreground social space.

**Table 2:** Correlation between Spatial Landscape Element Characteristics and Restorative Perception in Laoban hill.

Laoban Hill		Standardized Coefficient	T	Salience	95.0% Confidence Interval for Beta		Collinear Statistics	
		Beta			Upper Limit	Lower Limit	Tolerance	VIF
Being Away (R^2^ = 48.8%)	View probability of Rest Seat	0.443	3.158	0.004	0.382	1.805	0.999	1.001
	Gentle slope of the trail	−0.353	−2.501	0.019	−1.066	−0.104	0.985	1.015
	Ladder shade coverage	0.354	2.501	0.019	0.135	1.387	0.984	1.016
Extent (R^2^ = 45.2%)	Arbor species	−0.444	−2.903	0.007	−2.640	−0.451	0.907	1.103
	Cultural character of the landscape wall	0.353	2.155	0.041	0.029	1.243	0.789	1.267
Fascination (R^2^ = 21.0%)	Harmony of sculpture with the environment	0.458	2.726	0.011	0.211	1.489	1.000	1.000
Compatibility (R^2^ = 37.1%)	Flowering season	−0.381	−2.366	0.025	−0.516	−0.037	0.900	1.111
	Cultural character of the landscape wall	0.370	2.299	0.029	0.066	1.155	0.900	1.111

**Table 3:** Correlation between Spatial Landscape Element Characteristics and Restorative Perception in Jiuqu bridge.

Jiuqu Bridge		Standardized Coefficient	T	Salience	95.0% Confidence Interval for Beta		Collinear Statistics	
		Beta			Upper Limit	Lower Limit	Tolerance	VIF
Being Away (R^2^ = 48.8%)	Proximity between garden bridge and plants	−0.381	−2.183	0.038	0.041	1.277	1.000	1.000
Extent (R^2^ = 45.2%)	Proximity between garden bridge and water	−0.386	−2.216	0.035	−0.748	−0.029	1.000	1.000
Fascination (R^2^ = 21.0%)	State of the Lake Water	0.463	3.429	0.002	0.407	1.625	0.996	1.004
	Climbing plant species	−0.581	−3.745	0.001	−1.675	−0.488	0.753	1.327
	Layer of aquatic plants	−0.449	−2.896	0.008	−1.612	−0.273	0.754	1.326
Compatibility (R^2^ = 37.1%)	Landscape lamp post light color	−0.368	−2.490	0.019	−1.401	−0.134	0.974	1.027
	Landscape lamp post height	0.404	2.766	0.010	0.178	1.206	0.997	1.003
	Harmony of garden pavilion and environment	0.357	2.413	0.023	0.195	2.443	0.972	1.029

**Table 4:** Correlation between Spatial Landscape Element Characteristics and Restorative Perception in the ginkgo garden.

The Ginkgo Garden		Standardized Coefficient	T	Salience	95.0% Confidence Interval for Beta		Collinear Statistics	
		Beta			Upper Limit	Lower Limit	Tolerance	VIF
Being Away (R^2^ = 32.6%)	Arbor planting density	−0.424	−2.685	0.012	−1.103	−0.147	1.000	1.000
	Distribution of rest seats	−0.382	−2.420	0.023	−2.995	−0.247	1.000	1.000
Extent (R^2^ = 17.9%)	Distribution of rest seats	−0.424	−2.475	0.020	−4.538	−0.428	1.000	1.000
Fascination (R^2^ = 46.6%)	Clarity of boundary	0.582	4.057	0.000	0.865	2.635	0.962	1.040
	Harmony of landscape stone with the environment	0.489	3.409	0.002	0.498	2.002	0.962	1.040
Compatibility (R^2^ = 41.0%)	Clarity of boundary	0.493	3.315	0.003	0.509	2.164	0.988	1.012
	Probability of staying at the site	0.359	2.416	0.023	0.104	1.271	0.988	1.012

## Data Availability

Not applicable.

## Appendix A

**Table A1:** Campus Landscape Preference Factor Scales of Laoban hill.

Landscape Elements	Landscape Components		Selecting Preferred Landscape Element Features
Flowers	Plant Height	Low Floor (10–20 cm)	
		Middle layer (20–40 cm)	
		High Floor (40–60 cm)	
	Plant Color	simple color	
		colorful	
	Cool and Warm Colors	cool flower	
		Neutral flowers (white, black)	
		warm flowers	
	Flowering Season	Spring (February–April)	
		Summer (May–July)	
		Autumn (August–October)	
		Winter (November–January)	
Arbor	Plant Density	Lush	
		Medium	
		Sparse	
	Species	Evergreen tree	
		Deciduous tree	
	Ornamental Features	Flower viewing	
		Foliage	
		View dry	
		View branches	
	Plant Species	Less variety but uniform	
		variety	
	Plant Color	Simple color	
		Colorful	
	Plant Layer	Low-layer and simple	
		Multi-layer and rich	
Gentle Slope	Trail Slope	0–3°	
		3–10°	
		10–27°	
	Shade Coverage	High (>50%)	
		Low (<50%)	
Ladder	Step Width	Large (above 300 mm)	
		General (240 mm–300 mm)	
	Step Aspect Ratio	Large (large step width, low height)	
		General	
	Shade Coverage	High (>50%)	
		Low (<50%)	
Garden Pavilion	Harmony with the Environment	Highly integrated with the landscape environment, harmonious and unified	
		Obvious differences in the landscape environment	
	View Probability	Open on all sides with great views	
		A certain occlusion, applying frame, scene leakage, and other techniques	
	Culture	Engraved with poem couplets, famous quotes, etc.	
		No poems, couplets, famous quotes, etc.	
Landscape Stone	Size	Large Ornamental Stone (65–100 cm)	
		Medium Ornamental Stone (20–65 cm)	
		Small Ornamental Stone (5–20 cm)	
	Harmony with the Environment	Highly integrated with the landscape environment (harmonious and unified)	
		Obvious differences in the overall landscape environment	
	Culture	Engraved with poem couplets, famous quotes, etc.	
		No poems, couplets, famous quotes, etc.	
Cultural Card	Harmony with the Environment	Highly integrated with the landscape environment (harmonious and unified)	
		Obvious differences in overall landscape environment	
	Culture	Engraved with poem couplets, famous quotes, etc.	
		No poems, couplets, famous quotes, etc.	
Landscape Lamp Post	Street Light Height	High (above 4 m)	
		General (3 m–4 m)	
		Short (2.5 m–3 m)	
	Lighting Effects	Bright (no dark areas at night)	
		Dark (partially dark areas at night, strong privacy)	
	Light Color	Cold	
		Warm	
	Types	Many (two or more types, including street lights, lawn lights, decorative lights, etc.)	
		Less (street lights only)	
Garbage Can	Harmony with the Environment	Highly integrated with the landscape environment (harmonious and unified)	
		Obvious differences in the overall landscape environment	
	Distribution	Even distribution (easy to use)	
		Centralized distribution to create a simple landscape environment	
Rest Seat	High	Height (>48 cm)	
		General (38 cm–48 cm)	
		Low (<38 cm)	
	Harmony with the Environment	Highly integrated with the landscape environment (harmonious and unified)	
		Obvious differences in the overall landscape environment	
	View Probability	Open viewing interface and highly observable landscape environment	
		Blocked viewing interface, and strong privacy	
	Distribution	Even distribution (easy to use)	
		Centralized distribution to create a simple landscape environment	
Landscape Wall	Culture	Engraved with poem couplets, famous quotes, etc.	
		No poems, couplets, famous quotes, etc.	
	Location	Straight Ahead	
		Side Of Sight	
Sculpture	Size	Large Sculpture (above 3 m)	
		Medium Sculpture (1.5–3 m)	
		Small sculpture (below 1.5 m)	
	Harmony with the Environment	Highly integrated with the landscape environment (harmonious and unified)	
		Obvious differences in the overall landscape environment	
Behavioral Perception	Border Definition	Clear roads, boundaries, etc.	
		Blurred roads, borders, etc.	
	Probability of Stay	Long-term Stay	
		Short Stay	
		No Stop	
	Sense of Security	Back interface, transparent line of sight, and a strong sense of security	
		Enclosed on three sides, blocked line of sight, and strong privacy	
		No enclosure, transparent line of sight, a strong sense of security	
	Walking Comfort (pavement material)	Asphalt Road	
		Concrete Pavement	
		Brick Pavement	
		Natural Stone Pavement	
		Wooden Pavement	
		Gravel Road	
	Twists and Turns of Space	Many changes in opening and closing space and strong emotional ups and downs	
		Less opening and closing space changes, more stable emotions	

**Table A2:** Campus Landscape Preference Factor Scales of Jiuqu bridge.

Landscape Elements	Landscape Components		Selecting Preferred Landscape Element Features
Aquatic Plants	Proportion of Water Surface	High (above 50%)	
		Low (below 50%)	
	Flowering Season	Spring (February–April)	
		Summer (May–July)	
		Autumn (August–October)	
		Winter (November–January)	
	Plant Species	Less variety but uniform	
		Variety	
	Plant Color	Simple color	
		Colorful	
	Plant Layer	Low-layer and simple	
		Multi-layer and rich	
Shrub	Boundary Enclosure	Creates a private space	
		Creates open space	
	Plant Height	High (1.5–3 m)	
		Medium (0.5–1.5 m)	
		Low (below 0.5 m)	
	Plant Density	Lush	
		Medium	
		Sparse	
	Planting Method	Orphan	
		Group Planting	
		Planting	
	Plant Species	Less variety but uniform	
		Variety	
	Plant Color	Simple color	
		Colorful	
	Plant Layer	Low-layer and simple	
		Multi-layer and rich	
Arbor	Plant Height	High (above 9 m)	
		Medium (5–9 m)	
		Low (below 5 m)	
	Plant Density	Lush	
		Medium	
		Sparse	
	Planting Method	Orphan	
		Group Planting	
		Planting	
	Species	Evergreen Tree	
		Deciduous Tree	
	Ornamental Features	Flower viewing	
		Foliage	
		View dry	
		View branches	
	Plant Species	Less variety but uniform	
		Variety	
	Plant Color	Simple color	
		Colorful	
	Plant Layer	Low-layer and simple	
		Multi-layer and rich	
Climbing Plants	D/H of Road Width and Climbing Height	<1:1—(high borders, narrow roads)	
		≈1:1 (the height of the border is comparable to the width of the road)	
		>1:1 (low border, wide road)	
	Flowering Season	Spring (February–April)	
		Summer (May–July)	
		Autumn (August–October)	
		Winter (November–January)	
	Plant Species	Less variety but uniform	
		Variety	
	Plant Color	Simple color	
		Colorful	
	Plant Layer	Low-layer and simple	
		Multi-layer and rich	
Lake	Number of Aquatic (higher) animals	Many (more than 6)	
		less (1–5)	
	Aquatic (lower) Animal Characteristics	Have	
		None	
	Water State	Dynamic Water Feature	
		Static Water Feature	
Terrain	Flatness	Flat Terrain	
		Full Of Ups and Downs	
	Shade Coverage	High (>50%)	
		Low (<50%)	
Garden Pavilion	Harmony with the Environment	Highly integrated with the landscape environment (harmonious and unified)	
		Obvious differences in the landscape environment	
	View Probability	Open on all sides with great views	
		a certain occlusion, applying frame, scene leakage, and other techniques	
	Culture	Engraved with poem couplets, famous quotes, etc.	
		No poems, couplets, famous quotes, etc.	
Garden Bridge	Scale	Width (>2.2 m)	
		Suitable (1.1 m–2.2 m)	
		Narrow (<1.1 m)	
	Guardrail Height	High guardrail height, good safety	
		Guardrail height is suitable	
		Low guardrail height or no guardrail, high closeness to the environment	
	Harmony with the Environment	Highly integrated with the landscape environment, harmonious and unified	
		There are obvious differences in the overall landscape environment	
	Sense of Space	Open space, easy to see	
		constricted space, and concentrated line of sight	
	Route Settings	Straight and easy to navigate	
		Twisted for easy viewing	
	Culture	Engraved with poem couplets, famous quotes, etc.	
		No poems, couplets, famous quotes, etc.	
	Proximity with Plants	Tall (easy-to-touch plants)	
		General (can-touch plants)	
		Low (a certain distance from plants)	
	Proximity with Water	High (easy-to-touch the water)	
		General (can-touch the water)	
		Low, a certain distance from the water	
Landscape Stone	Size	Large Ornamental Stone (65–100 cm)	
		Medium Ornamental Stone (20–65 cm)	
		Small Ornamental Stone (5–20 cm)	
	Harmony with the Environment	Highly integrated with the landscape environment (harmonious and unified)	
		Obvious differences in overall landscape environment	
	Culture	Engraved with poem couplets, famous quotes, etc.	
		No poems, couplets, famous quotes, etc.	
Landscape Lamp Post	Street Light Height	High (above 4 m)	
		General (3 m–4 m)	
		Short (2.5 m–3 m)	
	Lighting Effects	Bright (no dark areas at night)	
		Dark (partially dark areas at night, strong privacy)	
	Light Color	Cold	
		Warm	
	Types	Many (two or more types, including street lights, lawn lights, decorative lights, etc.)	
		Less (street lights only)	
Garbage Can	Harmony with the Environment	Highly integrated with the landscape environment (harmonious and unified)	
		Obvious differences in the overall landscape environment	
	Distribution	Even distribution, easy to use	
		Centralized distribution to create a simple landscape environment	
Rest Seat	High	Height (>48 cm)	
		General (38 cm–48 cm)	
		Low (<38 cm)	
	Harmony with the Environment	Highly integrated with the landscape environment (harmonious and unified)	
		Obvious differences in the overall landscape environment	
	View Probability	open viewing interface, and highly observable landscape environment	
		blocked viewing interface, and strong privacy	
Behavioral Perception	Border Definition	Clear roads, boundaries, etc.	
		Blurred roads, borders, etc.	
	Probability of Stay	Long-term Stay	
		Short Stay	
		No Stop	
	Sense of Security	Back interface, transparent line of sight, and a strong sense of security	
		Enclosed on three sides, blocked line of sight, and strong privacy	
		No enclosure, transparent line of sight, a strong sense of security	
	Walking Comfort (pavement material) Border Definition	Asphalt Road	
		Concrete Pavement	
		Brick Pavement	
		Natural Stone Pavement	
		Clear roads, boundaries, etc.	
		Blurred roads, borders, etc.	
	Probability of Stay	Long-term Stay	
		Short Stay	

**Table A3:** Campus Landscape Preference Factor Scales of the ginkgo garden.

Landscape Elements	Landscape Components		Selecting Preferred Landscape Element Features
Lawn	Lawn Fineness	Rough (leaf width > 3 mm)	
		Delicate (leaf width 1.5–3 mm)	
	Height of Cut	Height (>8 cm)	
		Medium (4–8 cm)	
		Low (<4 cm)	
	Turf Purity Proportion	Pure Lawn	
		30% Of Other Grass and Flowers	
		60% Of Other Grass and Flowers	
Flowers	Plant Height	Low Floor (10–20 cm)	
		The middle layer (20–40 cm)	
		High Floor (40–60 cm)	
	Plant Color	Simple Color	
		Colorful	
	Cool and Warm Colors	Cool Flower	
		Neutral Flowers (white, black)	
		Warm Flowers	
	Flowering Season	Spring (February–April)	
		Summer (May–July)	
		Autumn (August–October)	
		Winter (November–January)	
Arbor	Branch Point Height	High (above 4 m)	
		Low (2.5 m–4 m)	
	Branch Uniformity	Tidy	
		Scattered	
	Plant Height	Height (9 m)	
		Medium (5–9 m)	
		Low (below 5 m)	
	Planting Density	Lush	
		Medium	
		Sparse	
	Species	Evergreen tree	
		Deciduous tree	
	Ornamental Features	Flower viewing	
		Foliage	
		View dry	
		View branches	
	Plant Species	Less variety but uniform	
		Variety	
	Plant Color	Simple color	
		Colorful	
	Plant Layer	Low layer and simple	
		Multi-layer and rich	
Terrain	Flatness	Flat Terrain	
		Full of Ups and Downs	
	Shade Coverage	High (>50%)	
		Low (<50%)	
Landscape Stone	Size	Large Ornamental Stone (65–100 cm)	
		Medium Ornamental Stone (20–65 cm)	
		Small Ornamental Stone (5–20 cm)	
	Harmony with the Environment	Highly integrated with the landscape environment (harmonious and unified)	
		Obvious differences in overall landscape environment	
	Culture	Engraved with poem couplets, famous quotes, etc.	
		No poems, couplets, famous quotes, etc.	
Rest Seat	High	Height (>48 cm)	
		General (38 cm–48 cm)	
		Low (<38 cm)	
	Harmony with the Environment	Highly integrated with the landscape environment (harmonious and unified)	
		Obvious differences in the overall landscape environment	
	View Probability	Open viewing interface, and highly observable landscape environment	
		Blocked viewing interface and strong privacy	
	Distribution	Even distribution (easy to use)	
		Centralized distribution to create a simple landscape environment	
Landscape Lamp Post	Street Light Height	High (above 4 m)	
		General (3 m–4 m)	
		Short (2.5 m–3 m)	
	Lighting Effects	Bright (no dark areas at night)	
		Dark (partially dark areas at night, strong privacy)	
	Light Color	Cold	
		Warm	
	Types	Many (two or more types, including street lights, lawn lights, decorative lights, etc.)	
		Less (street lights only)	
Behavioral Perception	Border Definition	Clear roads, boundaries, etc.	
		Blurred roads, borders, etc.	
	Probability of Stay	Long-term Stay	
		Short Stay	
		No Stop	
	Sense of Security	Back interface, transparent line of sight, and strong sense of security	
		Enclosed on three sides, blocked line of sight, and strong privacy	
		No enclosure, transparent line of sight, a strong sense of security	
	Walking Comfort (pavement material)	Asphalt Road	
		Concrete Pavement	
		Brick Pavement	
		Natural Stone Pavement	
		Wooden Pavement	
		Gravel Road	
	Twists and Turns of Space	Many changes in opening and closing space, and strong emotional ups and downs	
		Less space opening and closing changes, more stable emotions	
